# Assisted Reproductive Technology in Japan: A Summary Report for 2023 by the Committee on Professional Scientific Conduct and Clinical Ethics of the Japan Society of Obstetrics and Gynecology

**DOI:** 10.1002/rmb2.70008

**Published:** 2025-12-23

**Authors:** Yukiko Katagiri, Seung Chik Jwa, Akira Kuwahara, Takeshi Iwasa, Masanori Ono, Keiichi Kato, Hiroshi Kishi, Yoshimitsu Kuwabara, Fuminori Taniguchi, Miyuki Harada, Akira Iwase, Norihiro Sugino

**Affiliations:** ^1^ Department of Obstetrics and Gynecology, Faculty of Medicine Toho University Tokyo Japan; ^2^ Department of Obstetrics and Gynecology Jichi Medical University Tochigi Japan; ^3^ Department of Obstetrics and Gynecology, Graduate School of Biomedical Sciences Tokushima University Tokushima Japan; ^4^ Department of Obstetrics and Gynecology Tokyo Medical University Tokyo Japan; ^5^ Kato Ladies Clinic Tokyo Japan; ^6^ Department of Obstetrics and Gynecology The Jikei University School of Medicine Tokyo Japan; ^7^ Department of Obstetrics and Gynecology Nippon Medical School Tokyo Japan; ^8^ Department of Obstetrics and Gynecology Tottori University Faculty of Medicine Tottori Japan; ^9^ Department of Obstetrics and Gynecology, Graduate School of Medicine The University of Tokyo Tokyo Japan; ^10^ Department of Obstetrics and Gynecology Gunma University Graduate School of Medicine Maebashi Japan; ^11^ Yamaguchi University Graduate School of Medicine Ube Japan

**Keywords:** assisted reproductive technologies, fertility rate, in vitro fertilization, intracytoplasmic sperm injections, Japan

## Abstract

**Purpose:**

To summarize assisted reproductive technology (ART) data for 2023 collected through the Japan Society of Obstetrics and Gynecology registry.

**Methods and Results:**

625 out of 638 registered ART facilities took part in the study, documenting 561 664 treatment cycles and 85 048 newborns (+3.3% and +10.2% increases from 2022). The average age of women undergoing treatment was 37.3 years (standard deviation: 4.8); 205 986 cycles (36.7%) involved women aged ≥ 40 years. Among fresh cycles, oocyte retrieval was performed in 281 665 cycles, including 168 343 freeze‐all cycles (59.8%). A total of 2329 pregnancies and 3375 newborns resulted from in vitro fertilization and intracytoplasmic sperm injection cycles, yielding 1772 and 2502 newborns, respectively. The overall rates for single embryo transfer (SET) and singleton delivery were 80.5% and 96.5%. Frozen–thawed embryo transfer accounted for 271 361 cycles, resulting in 109 850 pregnancies and 80 774 newborns, with a SET rate of 84.7% and a singleton delivery rate of 96.3%.

**Conclusions:**

In 2023, the second year of insurance coverage, the registry recorded the highest numbers of treatment cycles and newborns. Effective registry systems planned for 2026 will enable comprehensive evaluation of emerging trends in Japanese ART practice.

## Graphical abstract

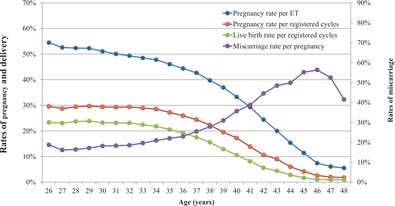

## 1 Introduction

Assisted reproductive technology (ART) has become an essential component of infertility treatment in Japan as declining fertility and delayed childbearing continue to shape demographic trends. Following the birth of Japan's first in vitro fertilization (IVF)‐conceived infant in 1983, ART utilization has expanded rapidly, and the country now performs some of the highest numbers of treatment cycles worldwide [1, 2].

The fertility rate in Japan has been decreasing steadily, with the total fertility rate reaching historic lows of 1.26 and 1.20 births per woman in 2022 and 2023 [3]. Given these declining birth rates and the increasing trend toward later childbearing, the contribution of ART to the overall birth rate in Japan continues to grow. Since April 2022, ART treatments have been covered by public health insurance [4], which has significantly increased access to fertility care [5].

To evaluate the effectiveness and safety of ART, and to understand the current state of ART implementation, it is essential to monitor national trends of use and its outcomes. The of Japan Society of Obstetrics and Gynecology (JSOG) ART registry was established to monitor these nationwide activities and has provided annual data describing clinical practice for nearly two decades. With Japan reporting its lowest‐ever total fertility rate in recent years and insurance coverage for many ART procedures beginning in 2022, it is increasingly important to document year‐to‐year changes in treatment volume and outcomes. The current report summarizes national ART activity for 2023 using data collected through the JSOG registry system [6].

## 2 Materials and Methods

### 2.1 Data Source and Study Population

The ART registry managed by the JSOG compiles comprehensive information from registered ART facilities throughout Japan. This registry data encompasses demographic and background characteristics such as infertility diagnoses, treatment information, and pregnancy and obstetric outcomes documented as cycle‐specific records [6]. This current descriptive analysis examined registered ART cycles conducted in 2023 with a cutoff date of 30 November 2024 and specifically evaluated the frequencies and characteristics of treatments performed, along with associated pregnancy outcomes.

### 2.2 ART Treatment Cycles

Data were collected, analyzed, and compared across years for each fertilization method—IVF, intracytoplasmic sperm injection (ICSI), and frozen–thawed embryo transfer (FET)—including the number of registered treatment cycles, oocyte retrievals, embryo transfer (ET) cycles, freeze‐all embryo or oocyte cycles, and the numbers of pregnancies and live births. Characteristics of registered cycles and pregnancy outcomes were summarized separately for fresh and FET cycles. Fresh cycle data were further categorized according to the fertilization method (IVF or ICSI using ejaculated/nonejaculated sperm).

### 2.3 Pregnancy Outcomes

Treatment results including clinical pregnancy (defined as confirmation of gestational sac in utero), live birth (defined as delivery of at least one live neonate after 22 weeks of gestation or later), and multiple pregnancy rates were evaluated. Pregnancy outcomes included ectopic or heterotopic pregnancy, artificial abortion, stillbirth, and fetal reduction. Finally, the treatment outcomes of pregnancy, live birth, miscarriage, and multiple pregnancy rates were evaluated based on patient age. Furthermore, the clinical outcomes of cycles using previously frozen oocytes were assessed.

### 2.4 Statistical Analysis

All analyses were conducted using the STATA MP statistical package, version 19.5 (Stata, College Station). Statistical testing was not conducted as this study focuses on descriptive analysis.

## 3 Results

In 2023, of the 638 registered ART facilities, 625 participated in the JSOG registry and, of these, 610 actually implemented ART treatment.

Table 1 summarizes the main trends in the numbers of registered cycles, egg retrievals, pregnancy, and neonate births categorized by IVF, ICSI, and FET cycles in Japan (2007–2023). In 2023, 561 664 cycles were registered for IVF, ICSI, and FET, and a total of 85 048 neonates were recorded in Japan, representing 3.3% and 10.2% increases from the previous year. Of note, the number of IVF cycles registered decreased by 1.7%, while ICSI cycles increased by 4.2% from the numbers reported in 2022.

**TABLE 1:** Trends in numbers of registered cycles, egg retrieval, pregnancy, and neonates according to IVF, ICSI, and frozen–thawed embryo transfer cycles, Japan, 2007–2023.

Year	Fresh cycles												FET cycles^c^			
	IVF^a^						ICSI^b^									
	No. of registered cycles	No. of egg retrieval	No. of freeze‐all cycles	No. of ET cycles	No. of cycles with pregnancy	No. of neonates	No. of registered cycles	No. of egg retrieval	No. of freeze‐all cycles	No. of ET cycles	No. of cycles with pregnancy	No. of neonates	No. of registered cycles	No. of ET cycles	No. of cycles with pregnancy	No. of neonates
2007	53 873	52 165	7626	28 228	7416	5144	61 813	60 294	11 541	34 032	7784	5194	45 478	43 589	13 965	9257
2008	59 148	57 217	10 139	29 124	6897	4664	71 350	69 864	15 390	34 425	7017	4615	60 115	57 846	18 597	12 425
2009	63 083	60 754	11 800	28 559	6891	5046	76 790	75 340	19 046	35 167	7330	5180	73 927	71 367	23 216	16 454
2010	67 714	64 966	13 843	27 905	6556	4657	90 677	88 822	24 379	37 172	7699	5277	83 770	81 300	27 382	19011
2011	71 422	68 651	16 202	27 284	6341	4546	102 473	100 518	30 773	38 098	7601	5415	95 764	92 782	31 721	22 465
2012	82 108	79 434	20 627	29 693	6703	4740	125 229	122 962	41 943	40 829	7947	5498	119 089	116 176	39 106	27 715
2013	89 950	87 104	25 085	30 164	6817	4776	134 871	134 871	49 316	41 150	8027	5630	141 335	138 249	45 392	32 148
2014	92 269	89 397	27 624	30 414	6970	5025	144 247	141 888	55 851	41 437	8122	5702	157 229	153 977	51 458	36 595
2015	93 614	91 079	30 498	28 858	6478	4629	155 797	153 639	63 660	41 396	8169	5761	174 740	171 495	56 888	40 611
2016	94 566	92 185	34 188	26 182	5903	4266	161 262	159 214	70 387	38 315	7324	5166	191 962	188 338	62 749	44 678
2017	91 516	89 447	36 441	22 423	5182	3731	157 709	155 758	74 200	33 297	6757	4826	198 985	195 559	67 255	48 060
2018	92 552	90 376	38 882	20 894	4755	3402	158 859	157 026	79 496	29 569	5886	4194	203 482	200 050	69 395	49 383
2019	88 074	86 334	40 561	17 345	4002	2974	154 824	153014	83 129	24 490	4789	3433	215 203	211 758	74 911	54 188
2020	82 883	81 286	42 530	13 362	3094	2282	151 732	150 082	87 697	19 061	3626	2596	215 285	211 914	76 196	55 503
2021	88 362	86 901	42 016	13 219	3115	2268	170 350	168 659	86 992	19 740	3875	2850	239 428	236 211	87 174	64 679
2022	91 402	89 807	49 433	12 211	3007	2183	187 816	185 489	108 814	19 299	3878	2822	264 412	260 101	98 348	72 201
2023	89 854	88 378	52 317	9209	2329	1772	195 657	193 287	116 026	15 534	3375	2502	276 153	271 361	109 850	80 774

Abbreviations: ET, embryo transfer; FET, frozen‐thawed embryo transfer; ICSI, intracytoplasmic sperm injection; IVF, in vitro fertilization.

^a^ Including gamate intrafallopian transfer.

^b^ Including Split‐ICSI cycles.

^c^ Including cycles using frozen–thawed oocyte.

In contrast with 2022, the number of freeze‐all IVF and ICSI cycles increased by 5.8% and 6.6%, respectively. The number of neonates born by IVF‐ET cycles was 1772, and 2502 by ICSI, representing significant decreases (18.8% and 11.3%) from the previous year. The continuously increasing trend seen for FET cycles since 2007 was maintained in 2023, with a 4.4% increase. The number of registered FET cycles was 276 153, with 109 850 pregnancies and 80 774 neonates.

**FIGURE 1:**
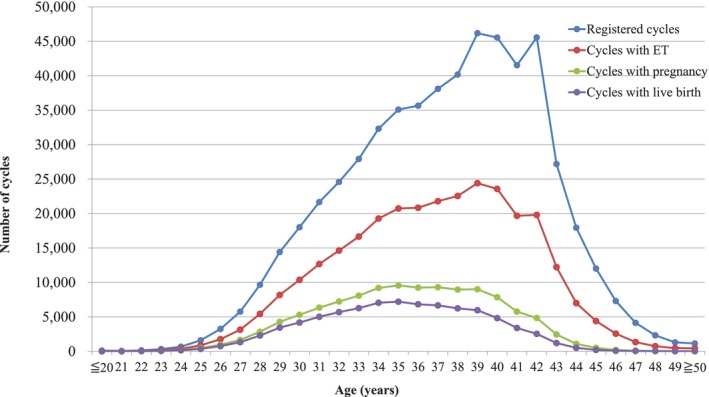
Distribution of maternal age from all registered cycles, cycles for ET, cycles leading to pregnancy, and cycles leading to live births in 2023. Adapted from the Japan Society of Obstetrics and Gynecology ART Databook 2023 (https://www.jsog.or.jp/activity/art/2023_JSOG‐ART.pdf). ET, embryo transfer.

Figure 1 shows the age distributions for all registered cycles and different subgroups of cycles for ET, pregnancy, and live births in 2023. The mean patient age for registered cycles was 37.3 years (standard deviation [SD] ± 4.8); the mean age for pregnancy and live birth cycles was 35.5 years (SD ± 4.3) and 35.1 years (SD ± 4.2), respectively. In 2023, 36.7% of ART cycles (205 986 cycles) registered were undertaken for women aged 40 years or over.

### 3.1 Treatment and Pregnancy Outcomes

The detailed characteristics and treatment outcomes of registered fresh cycles are shown in Table 2. In 2023, 82 634 IVF cycles, 35 457 split‐ICSI cycles, 157 943 ICSI cycles using ejaculated spermatozoa, 2257 ICSI cycles using testicular sperm extraction (TESE), 4014 cycles for oocyte freezing, and 3206 other cycles were registered. In total, 281 665 cycles resulted in oocyte retrieval, of which 168 343 (59.8%) were freeze‐all cycles. The pregnancy rate was 25.3% per ET cycle of IVF, and 20.8% for ICSI using ejaculated spermatozoa. The total single ET rate was 80.5%, and the pregnancy rate following a single ET cycle was 23.9%. Live birth rates per ET were 18.6% for IVF, 20.4% for split‐ICSI, 14.7% for ICSI using ejaculated spermatozoa, and 14.1% for ICSI with TESE. There were 5421 singleton pregnancies and 3979 singleton live births. In 2023, 4014 cycles for oocyte freezing were registered, and 3980 oocyte retrievals were conducted. Of these, 3679 cycles led to successfully frozen oocytes. The singleton pregnancy rate was 96.6%, and the singleton live birth rate was 96.5%.

**TABLE 2:** Characteristics and treatment outcomes of registered fresh cycles in assisted reproductive technology, Japan, 2023.

Variables	IVF	Split	ICSI		Frozen oocyte	Others^a^	Total
			Ejaculated sperm	TESE			
No. of registered cycles	82 634	35 457	157 943	2257	4014	3206	285 511
No. of egg retrievals (0 or more)	81 214	35 175	155 857	2255	3980	3184	281 665
No. of fresh ET cycles (1 or more)	9034	2197	13 067	270	0	175	24 743
No. of freeze‐all cycles	47 026	28 440	86 215	1371	3679	1612	168 343
No. of cycles with pregnancy	2281	609	2719	47	0	48	5704
Pregnancy rate per ET	25.3%	27.7%	20.8%	17.4%		27.4%	23.1%
Pregnancy rate per egg retrieval	2.8%	1.7%	1.7%	2.1%		1.5%	2.0%
Pregnancy rate per egg retrieval excluding freeze‐all cycles	6.7%	9.0%	3.9%	5.3%		3.1%	5.0%
SET cycles	7636	1881	10 072	167		153	19 909
Pregnancy following SET cycles	1997	543	2135	40		44	4759
Rate of SET cycles	84.5%	85.6%	77.1%	61.9%		87.4%	80.5%
Pregnancy rate following SET cycles	26.2%	28.9%	21.2%	24.0%		28.8%	23.9%
Miscarriages	514	135	718	8		9	1384
Miscarriage rate per pregnancy	22.5%	22.2%	26.4%	17.0%		18.8%	24.3%
Singleton pregnancies^b^	2182	576	2570	46		47	5421
Multiple pregnancies^b^	59	18	110	0		1	188
Twin pregnancies^b^	58	18	110	0		1	187
Triplet pregnancies^b^	1	0	0	0		0	1
Quadruplet pregnancies^b^	0	0	0	0		0	0
Multiple pregnancy rate^b^	2.6%	3.0%	4.1%	0.0%		2.1%	3.4%
Live births	1680	449	1919	38		39	4125
Live birth rate per ET	18.6%	20.4%	14.7%	14.1%		22.3%	16.7%
Total number of neonates	1722	463	2001	38		50	4274
Singleton live births	1636	435	1831	38		39	3979
Twin live births	43	14	85	0		0	142
Triplet live births	0	0	0	0		0	0
Pregnancy outcomes							
Ectopic pregnancies	22	9	33	1		0	65
Heterotopic pregnancy	0	0	2	0		0	2
Artificial abortions	13	2	10	0		0	25
Still births	2	0	6	0		0	8
Fetal reductions	0	1	0	0		0	1
Cycles with unknown pregnancy outcomes	33	8	27	0		0	68

Abbreviations: ET, embryo transfer; GIFT, gamate intrafallopian transfer; ICSI, intracytoplasmic sperm injection; IVF‐ET, in vitro fertilization–embryo transfer; SET, single embryo transfer; TESE, testicular sperm extraction.

^a^ Others include ZIFT.

^b^ Singleton, twin, triplet, and quadruplet pregnancies were defined according to the number of gestational sacs in utero.

Table 3 summarizes the characteristics and treatment outcomes of FET cycles. In 2023, a total of 275 763 cycles were registered. Of these, 273 914 were registered as FET cycles. Of the latter, 269 551 FETs were conducted. With a pregnancy rate of 40.5%, FET cycles resulted in 109 119 pregnancies. FET cycles resulted in 28 464 miscarriages. The miscarriage rate per pregnancy was 26.1%, and the live birth rate per FET increased to 28.8% from 27.0% observed in 2022. The single ET rate was 84.7%, somewhat lower than in 2022 (85.3%), resulting in a slightly increased pregnancy rate of 41.1% from 38.8% in 2022. The rate of singleton pregnancies was 96.2%, and the rate of singleton live births was 96.3%.

**TABLE 3:** Characteristics and treatment outcomes of frozen cycles in assisted reproductive technology, Japan, 2023.

Variables	FET	Others^a^	Total
No. of registered cycles	273 914	1849	275 763
No. of FET	269 551	1602	271 153
No. of cycles with pregnancy	109 119	680	109 799
Pregnancy rate per FET	40.5%	42.5%	40.5%
SET cycles	228 392	1315	229 707
Pregnancy following SET cycles	93 955	565	94 520
Rate of SET cycles	84.7%	82.1%	84.7%
Pregnancy rate following SET cycles	41.1%	43.0%	41.2%
Miscarriages	28 464	147	28 611
Miscarriage rate per pregnancy	26.1%	21.6%	26.1%
Singleton pregnancies^b^	104 072	635	104 707
Multiple pregnancies^b^	4128	35	4163
Twin pregnancies^b^	4055	34	4089
Triplet pregnancies^b^	67	1	68
Quadruplet pregnancies^b^	6	0	6
Multiple pregnancy rate^b^	3.8%	5.2%	3.8%
Live births			
Live birth rate per FET	28.8%	31.8%	28.8%
Total number of neonates	80 196	543	80 739
Singleton live births	74 692	475	75 167
Twin live births	2713	34	2747
Triplet live births	26	0	26
Pregnancy outcomes			
Ectopic pregnancies	477	1	478
Intrauterine pregnancies coexisting with ectopic pregnancy	20	0	20
Artificial abortions	551	4	555
Still births	277	7	284
Fetal reduction	33	0	33
Cycles with unknown pregnancy outcomes	1389	5	1394

Abbreviations: FET, frozen–thawed embryo transfer; SET, single embryo transfer.

^a^ Including cycles using frozen–thawed oocyte.

^b^ Singleton, twin, triplet, and quadruplet pregnancies were defined according to the number of gestational sacs in utero.

### 3.2 Outcomes by Patient Age

Table 4 shows the treatment outcomes of registered cycles by patient age in Japan in 2023. The pregnancy rate per ET exceeded 50% for women aged between 25 and 31 years. Gradual decreases in pregnancy rates per ET were observed with increasing maternal age, starting at age 27 years. Rates fell below 30% for women aged ≧ 41 years, below 20% among women aged > 43 years, and below 10% for women aged > 45 years. The miscarriage rates tended to be below 20% for all women aged under 34 years and increased gradually with increasing maternal age. Women in their early forties had miscarriage rates generally between 35.6% and 49.9%, while women in their mid‐forties had miscarriage rates over 55%. The live birth rate per registered cycle was the highest for women aged 29 years (23.8%). Rates declined sharply to below 15.0% at 39 years of age and below 10.0% among women ≧ 41 years of age.

**TABLE 4:** Treatment outcomes of registered cycles according to patients' age, Japan, 2023.

Age (years)	No. of registered cycles	No. of ET cycles	Pregnancy	Multiple pregnancies^a^	Miscarriage	Live birth	Pregnancy rate per ET (%)	Pregnancy rate per registered cycles (%)	Live birth rate per registered cycles (%)	Miscarriage rate per pregnancy (%)	Multiple pregnancy rate^a^ (**%**)
Under 20s	103	14	5	0	1	4	35.7	4.9	3.9	20.0	0.0
21	59	20	9	0	2	7	45.0	15.3	11.9	22.2	0.0
22	134	56	26	0	3	23	46.4	19.4	17.2	11.5	0.0
23	311	163	78	1	15	60	47.9	25.1	19.3	19.2	1.3
24	667	361	180	3	31	144	49.9	27.0	21.6	17.2	1.7
25	1597	864	470	16	105	343	54.4	29.4	21.5	22.3	3.4
26	3233	1757	957	29	179	754	54.5	29.6	23.3	18.7	3.0
27	5754	3144	1653	62	267	1326	52.6	28.7	23.0	16.2	3.8
28	9664	5429	2842	99	466	2287	52.3	29.4	23.7	16.4	3.5
29	14 411	8184	4277	153	732	3437	52.3	29.7	23.8	17.1	3.6
30	18 016	10 370	5295	189	961	4181	51.1	29.4	23.2	18.1	3.6
31	21 665	12 666	6342	207	1160	5006	50.1	29.3	23.1	18.3	3.3
32	24 603	14 631	7224	292	1339	5685	49.4	29.4	23.1	18.5	4.0
33	27 917	16 653	8076	303	1584	6261	48.5	28.9	22.4	19.6	3.8
34	32 314	19 266	9206	327	1927	7039	47.8	28.5	21.8	20.9	3.6
35	35 097	20 744	9559	391	2099	7206	46.1	27.2	20.5	22.0	4.1
36	35 664	20 835	9251	367	2125	6832	44.4	25.9	19.2	23.0	4.0
37	38 106	21 788	9298	354	2365	6667	42.7	24.4	17.5	25.4	3.8
38	40 182	22 545	8957	376	2489	6231	39.7	22.3	15.5	27.8	4.2
39	46 181	24 409	9017	323	2790	5964	36.9	19.5	12.9	30.9	3.6
40	45 544	23 578	7847	315	2796	4819	33.3	17.2	10.6	35.6	4.0
41	41 538	19 672	5763	260	2232	3383	29.3	13.9	8.1	38.7	4.5
42	45 572	19 803	4840	161	2155	2531	24.4	10.6	5.6	44.5	3.3
43	27 193	12 221	2444	78	1185	1191	20.0	9.0	4.4	48.5	3.2
44	17 945	7006	1077	27	537	507	15.4	6.0	2.8	49.9	2.5
45	12 012	4388	500	8	276	202	11.4	4.2	1.7	55.2	1.6
46	7297	2563	190	5	107	75	7.4	2.6	1.0	56.3	2.6
47	4127	1345	82	2	43	38	6.1	2.0	0.9	52.4	2.4
48	2323	741	41	2	17	22	5.5	1.8	0.9	41.5	4.9
49	1300	459	26	2	10	14	5.7	2.0	1.1	38.5	7.7
Over 50s	1135	429	22	1	11	11	5.1	1.9	1.0	50.0	4.5

Abbreviation: ET, embryo transfer.

^a^ Multiple pregnancies were defined according to the number of gestational sacs in utero.

**FIGURE 2:**
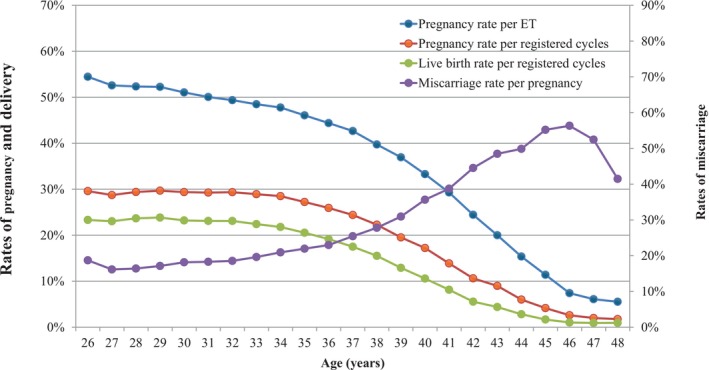
Pregnancy, live birth, and miscarriage rates according to patient age in all registered cycles 2023. Adapted from the Japan Society of Obstetrics and Gynecology ART Databook 2023 (https://www.jsog.or.jp/activity/art/2023_JSOG‐ART.pdf). ET, embryo transfer.

Figure 2 shows the rates of pregnancy, live birth, and miscarriage by patient age in all registered cycles in 2023. Of note, the pregnancy rate per ET was over 50% at ages 25 and 31 and generally above 45% under 36 years. There was then a progressive decline from that point, which became even more marked beyond the age of 40 years, similar to that reported in the previous year. Similar trends were observed for pregnancy and live birth rates, with progressive declines starting as early as 35 years of age. Conversely, miscarriage rates gradually increased from the early thirties up to 38 years of age and increased rapidly thereafter until the late forties.

### 3.3 Treatment Outcomes for FET Cycles Using Frozen–Thawed Oocytes

Table 5 shows the primary treatment outcomes of embryo transfers using frozen–thawed oocytes in Japan in 2023. In 2023, 390 cycles using frozen–thawed oocytes were registered in Japan, of which 208 FETs were implemented. Fifty‐one pregnancies were achieved, with a pregnancy rate per FET of 24.5% and a live birth rate of 16.4%. The miscarriage rate per pregnancy was 27.5%.

**TABLE 5:** Treatment outcomes of embryo transfers using frozen–thawed oocyte in assisted reproductive technology, Japan, 2023.

Variables	Embryo transfer using frozen–thawed oocyte
No. of registered cycles	390
No. of ET	208
No. of cycles with pregnancy	51
Pregnancy rate per ET	24.5%
SET cycles	117
Pregnancy following SET cycles	31
Rate of SET cycles	56.3%
Pregnancy rate following SET cycles	26.5%
Miscarriages	14
Miscarriage rate per pregnancy	27.5%
Singleton pregnancies^a^	47
Multiple pregnancies^a^	2
Twin pregnancies^a^	2
Triplet pregnancies^a^	0
Quadruplet pregnancies^a^	0
Multiple pregnancy rate^a^	4.1%
Live births	34
Live birth rate per ET	16.4%
Total number of neonates	35
Singleton live births	33
Twin live births	1
Triplet live births	0
Pregnancy outcomes	
Ectopic pregnancies	0
Intrauterine pregnancies coexisting with ectopic pregnancy	0
Artificial abortions	1
Still births	0
Fetal reduction	0
Cycles with unknown pregnancy outcomes	0

Abbreviations: ET, embryo transfer; SET, single embryo transfer.

^a^ Singleton, twin, triplet, and quadruplet pregnancies were defined according to the number of gestational sacs in utero.

## 4 Discussion

We described the characteristics and outcomes of ART cycles registered in the Japanese ART registry system during 2023 and compared the present results with those from 2022 [6] and previous years [7, 8, 9]. The main findings of the Japanese ART registry in 2023 were as follows: in 2023, 561 664 cycles were registered; a total of 85 048 neonate births were recorded.

In 2023, there were modest increases in total ART cycles (3.3% increase) and neonates (10.2% increase), with 561 664 registered cycles and 85 048 neonates born. IVF cycles decreased by 1.7%, while ICSI cycles increased by 4.2%. Freeze‐all cycles accounted for 59.8% of cycles with oocyte retrieval, resulting in significant decreases of 18.8% and 11.3% in neonates born from IVF‐ET and ICSI‐ET cycles, respectively. FET cycles increased by 4.4%, with 276 153 registered cycles. A total of 205 986 cycles (36.7%) were for women aged 40 years or over. The total single ET and singleton pregnancy rates for fresh cycles were 80.5% and 96.6%, respectively, and the singleton live birth rate was 96.5%. For frozen cycles, the single ET rate was 84.7%. The rates of singleton pregnancies and singleton live births were 96.2% and 96.3%, respectively. Notably, 2023 marked the second year of public health insurance coverage for ART, achieving the highest number of treatment cycles and neonates born in the registry's history.

The pregnancy rate per FET cycle increased from 37.9% in 2022 to 40.5% in 2023, continuing the upward trend observed in recent years. This improvement may be attributed to two key factors. First, the introduction of insurance coverage in April 2022, which limits the number of embryo transfers to six for women under 40 and three for women aged 40–42, may have encouraged more careful selection of embryos with higher implantation potential for transfer. Second, the clinical implementation of preimplantation genetic testing for aneuploidy (PGT‐A) has likely contributed to improved pregnancy outcomes per transfer. Following a nationwide clinical study by the JSOG published in 2023, which demonstrated favorable pregnancy outcomes with PGT‐A in patients with recurrent implantation failure, recurrent pregnancy loss, or chromosomal structural rearrangement, PGT‐A was introduced into clinical practice in Japan in 2022 [10]. The year 2023 marks the second year of PGT‐A availability. Recent evidence suggests that the effectiveness of PGT‐A is age‐dependent. A large‐scale study using the Society for Assisted Reproductive Technology database showed that cumulative live birth with PGT‐A was significantly lower in individuals aged < 35 years (risk ratio: 0.96; 95% CI: 0.93–0.99) but significantly higher in those aged 38–40 years (risk ratio: 1.14; 95% CI: 1.07–1.20) compared with no PGT‐A [11]. In response to accumulating evidence on age‐specific effectiveness [11, 12], the JSOG expanded the indications for PGT‐A in 2025 to include couples with advanced women's age, even in their first IVF cycle. Future evaluation of this expanded indication through registry‐based analysis will be essential to assess its impact on treatment outcomes.

In 2023, the number of multiple pregnancies increased from the previous year; the number of multiple pregnancies based on gestational sacs was 4353, which was significantly higher (+32.8% increase) compared with 3203 in 2022. A recent study utilizing ART registry data reported that in 2022, the first year after insurance coverage introduction, the multiple embryo transfer rate significantly decreased from 15.4% to 15.0% (*p* < 0.001), while the multiple pregnancy rate remained similar (2.98% vs. 3.05%; *p* = 0.17). However, the absolute number of multiple pregnancies reached 3209, a level equivalent to that in 2007 before the single embryo transfer guidelines were introduced by the JSOG [13]. The study also demonstrated that the proportion of blastocyst transfers in multiple embryo transfers increased significantly across most age strata in FET cycles. The current 2023 data showed a further increase in the absolute number of multiple pregnancies, reaching the highest level in the registry's history. This trend may be partly attributed to the increased utilization of blastocyst‐stage embryos in multiple embryo transfers, as blastocysts have higher implantation potential compared to cleavage‐stage embryos [14, 15]. Given these concerning trends, detailed monitoring of single embryo transfer rates and comprehensive evaluation of the impact of the number of transferred embryos on perinatal outcomes are essential to ensure the continued safety of ART practice in Japan. Furthermore, investigation into the extent to which the increase in ART‐associated multiple pregnancies burdens perinatal healthcare resources is warranted.

This study has several strengths and limitations. The main strength is that registered ART facilities nationwide are required to register their treatment cycles, leading to exceptionally high reporting compliance (98.0% facility participation in 2023). The standardization of procedures and definitions for cycle‐specific information across registered ART facilities has reduced reporting bias. Furthermore, the comprehensive data collection including pregnancy outcomes enables evaluation of policy impacts and assessment of treatment safety in real‐world clinical practice.

However, several limitations exist. The cycle‐specific nature of the registry prevents identification of women with multiple deliveries during study periods, potentially affecting clustering analysis within individuals. Additionally, some data for which collection is not standardized, such as background information including previous surgical history, socioeconomic status, and lifestyle factors, may be incomplete or missing. The registration system has not been updated since 2007, and participating ART facilities are required to register cycle‐specific information manually one‐by‐one through on‐line, which is presumed to be quite time‐consuming and may lead to data input errors. Furthermore, the registry has not been effectively utilized from patients' perspective.

To address these limitations, the JSOG has established a working group to develop effective registry utilization since 2024. From 2026, a batch registration system will be implemented to enable time‐saving registration [16]. Additionally, the working group is updating data collection items to include ovarian reserve markers such as anti‐Müllerian hormone and detailed information on PGT. Furthermore, prediction models for live birth and pregnancy rates will be developed. These developments aim to enhance both data quality and patient‐centered applications of the registry in the near future.

In conclusion, in 2023, the second year following the introduction of public health insurance coverage for ART in Japan, the registry documented 561 664 treatment cycles and 85 048 neonates born, representing the highest numbers in the registry's history. While single embryo transfer rates remained consistently high for both fresh and frozen cycles with favorable singleton pregnancy and live birth rates, a concerning trend emerged with a substantial increase in multiple pregnancies from the previous year. The pregnancy rate per FET cycle showed improvement, and factors such as embryo grade and the use of PGT‐A may have contributed to this trend. To enable detailed analysis of such factors and address current limitations, the JSOG is implementing a new registration system from 2026, including batch registration capabilities and expanded data collection items. These enhancements will enable more detailed and comprehensive evaluation of ART practice in Japan, facilitating evidence‐based policy development and quality improvement initiatives.

## Ethics Statement

All procedures were performed according to the ethical standards of the relevant committees on human experimentation (institutional and national), as well as the Helsinki Declaration of 1964 and its later amendments. This report contains no studies performed by any authors that included animals.

## Conflicts of Interest

The authors have no conflicts of interest to disclose about the present work. “Seung Chik, Jwa,” “Akira, Iwase,” and “Takeshi, Iwasa,” are Editorial Board members of *Reproductive Medicine and Biology* and coauthors of this article. To minimize bias, they were excluded from all editorial decision‐making related to the acceptance of this article for publication.

## Data Availability

The data that support the findings of this study are available on request from the corresponding author. The data are not publicly available due to privacy or ethical restrictions.
